# Supplementary material for: The neurodevelopmental spectrum of CASK-related disorder
Source: J Neurodev Disord. 2025 Oct 2;17:60. doi: 10.1186/s11689-025-09643-3 (PMC12492526; doi:10.1186/s11689-025-09643-3)
Supplement: Supplementary file 1 — Supplementary Material 1. [file 11689_2025_9643_MOESM1_ESM.docx]

# Supplementary Materials for:

# The neurodevelopmental spectrum of *CASK*-related disorder

Jessica Martin^1^, Alkistis Mavrogalou-Foti^1^, Josefine Eck^1^, Laura Hattersley^2^, Kate Baker^1, 3, 4^

^1^ MRC Cognition and Brain Sciences Unit, University of Cambridge

^2^ CASK Research Foundation

^3^ Department of Medical Genetics, University of Cambridge

^4^ Department of Pathology, University of Cambridge

Contents

[Supplementary Materials for: 1](#_Toc203494427)

[The neurodevelopmental spectrum of *CASK*-related disorder 1](#_Toc203494428)

[**Supplementary Table 1.** Neurodevelopmental Questionnaire Measures. 2](#_Toc203494429)

[**Supplementary Table 3.** Current medication use in the BINGO *CASK*-related disorder group. 3](#_Toc203494430)

[**Supplementary Table 4.** Correlations in the BINGO *CASK*-related disorder group. 4](#_Toc203494431)

[**Supplementary Table 5a.** Impact of Epilepsy on Adaptive Ability: Regression Assumptions 5](#_Toc203494432)

[**Supplementary Table 5b.** Impact of Epilepsy on Adaptive Ability: Regression Results 5](#_Toc203494433)

[**Supplementary Table 6a.** Impact of Epilepsy and Age on Adaptive Ability: Regression Assumptions 5](#_Toc203494434)

[**Supplementary Table 6b.** Impact of Epilepsy and Age on Adaptive Ability: Regression Results 5](#_Toc203494435)

[**Supplementary Table 7.** Summary of characteristics according to MRI abnormality within the BINGO *CASK*-related disorder group. 6](#_Toc203494436)

[**References** 7](#_Toc203494437)

## **Supplementary Table 1.** Neurodevelopmental Questionnaire Measures.

| **Questionnaire** | **Description** | **Scoring Procedure** |
| --- | --- | --- |
| The Vineland Adaptive Behaviour Scales (VABS) (Third Edition, Parent/Caregiver Form, Vineland-3; 1) | The VABS is a standardised assessment of global adaptive ability, comprised of three subdomains (communication, daily living skills, and socialisation) and a separate measure of motor skills. | Raw VABS composite and subdomain scores were scaled according to published norms. For ease of interpretation, scores on the VABS composite were reversed, such that higher scores represent greater difficulties across measures. The VABS composite was categorised as ‘normal’, ‘mild’, ‘moderate’, ‘severe’ or ‘profound’, and was used here as a proxy measure of severity of developmental delay or intellectual disability (DD/ID). |
| Developmental Behaviour Checklist (DBC2; 2) | The DBC2 is a standardised measure of emotional-behavioural difficulties in individuals with ID. | T-scores for the total behavioural problems score (TBPS) and all 5 treatment subscales (disruptive/antisocial behaviour, self-absorbed behaviour, social relating, communication disturbance, and anxiety) were generated using the Australian norms. T-scores were not stratified for severity of ID due to missing VABS composite scores. |
| Social Responsiveness Scale (SRS-2; 3) | The SRS-2 is a screening tool designed to assess autism characteristics in the general population, and is comprised of 5 subscales (social awareness, social cognition, social communication, social motivation, and restricted interests and repetitive behaviour). | SRS-2 raw scores were converted to age- and sex-appropriate T-scores based on published norms. |
| Repetitive Behaviour Questionnaire (RBQ; 4) | The RBQ is a measure of repetitive behaviour frequency across 5 subscales (stereotyped behaviour, compulsive behaviour, insistence on sameness, restricted preferences, and repetitive use of language). | Scores on the RBQ were calculated using the verbal scoring approach and missing items were scored as 1 (never). |
| Flemish Cerebral Visual Impairment Questionnaire (FCVIQ; 5) | The FCVIQ is a measure of behaviours associated with CVI and its impact on everyday functioning. | The analysis here focused on total scores. For descriptive purposes, we generated the Sum Score described by Ortibus et al. (5). Using this score, an individual is considered at risk of CVI if they display a minimum of one behaviour in at least four of the FCVIQ domains. We also generated scores for the five factors identified by Ben Itzhak et al. (6) (object and face processing impairments, visual (dis)interest, clutter and distance viewing impairments, moving in space impairments, and anxiety-related behaviours), which we scored according to the method described by Crotti et al. (7). Higher scores on all FCVIQ measures reflect greater impairment. |
| Challenging Behaviour Questionnaire (CBQ; 8) | The CBQ is a measure of the presence and severity of behaviours that challenge (BtC), including self-injury, physical aggression, property destruction**,** and stereotypic behaviour. | The proportion of participants displaying each category of challenging behaviour was generated from parent-reported data indicating the presence of each category of behaviour in the past month on a yes/no basis. Severity scores for each category of challenging behaviour were calculated out of 14 based on the duration, frequency, severity of behaviour. Higher scores represent greater severity. This scoring approach is consistent with Bissell et al. (9). |
| Short Sensory Profile – 2^nd^ Edition (SSP-2; 10) | The SSP-2 measures sensory-related behaviours in daily life across four patterns of sensory processing - Seeking/Seeker, Avoiding/Avoider, Sensitivity/Sensor, and Registration/Bystander. These patterns are theorised to arise from the relationships between an individual’s neurological threshold for sensory stimulation, and self-regulation strategies in Dunn’s Sensory Processing Model (11). The SSP-2 also includes a total sensory and behavioural score. | Raw l subscale scores were calculated and classified according published norms. These scores were classified as “much less than others”, “less than others”, “just like the majority of others”, “more than others”, and “much more than others”, based on a bell curve normed distribution of scores from a sample of children without disabilities. |

## **Supplementary Table 3.** Current medication use in the BINGO *CASK*-related disorder group.

|  | **N*** | **%** | **Medications represented across the group** |
| --- | --- | --- | --- |
| At least one regular medication | 17/28 | 61% |  |
| At least one anti-seizure medication | 7/28  of which more than one 3/7 | 25% | Nitrazepam, lamotrigine, sodium valproate, levetiracetam, clobazam, topimarate, cannabidiol |
| At least one psychoactive medication | 3/28 | 11% | Fluoxetine, buspirone, guanfacine, trazodone |
| Medication for gastro-oesophageal reflux | 7/28 | 25% | Omeprazole, lansoprazole, erythromycin |
| Medication for constipation | 6/28 | 21% | Macrogol, senna, sodium citrate enema, polyethylene glycol |
| Medication for sleep difficulties | 8/28 | 29% | Melatonin, clonidine, chloral hydrate |
| Medication for muscle tightness | 3/28 | 11% | Baclofen |
| Medication for excessive saliva production | 4/28 | 14% | Hyoscine, glycopyrronium |

*Parent-report information about current medication use was available for 28/31 BINGO participants.

## **Supplementary Table 4.** Correlations in the BINGO *CASK*-related disorder group.

|  | Age | Sex | VABS Composite | DBC2 Total | SRS-2 Total | RBQ Total | CVI Total |
| --- | --- | --- | --- | --- | --- | --- | --- |
| Sex | τ = -0.19, *p* = 0.22, *p.adj* = 0.39 |  |  |  |  |  |  |
| VABS Composite | *r_s_* = 0.78, ***p* < .01**, ***p.adj* < .01** | *r_pb_* = -0.21, *p* = 0.29, *p.adj* = 0.48 |  |  |  |  |  |
| DBC2 Total | τ = -0.03, *p* = 0.82, *p.adj* = 0.84 | *r_pb_* = -0.07, *p* = 0.70, *p.adj* = 0.75 | *r* = -0.33, *p* = 0.09, *p.adj* = 0.24 |  |  |  |  |
| SRS-2 Total | τ = 0.20, *p* = 0.16, *p.adj* = 0.32 | *r_pb_* = -0.13, *p* = 0.51, *p.adj* = 0.62 | *r* = 0.15, *p* = 0.45, *p.adj* = 0.62 | *r* = 0.38, *p* = 0.05, *p.adj* = 0.22 |  |  |  |
| RBQ Total | τ = 0.09, *p* = 0.49, *p.adj* = 0.62 | τ = -0.32, ***p* = 0.04**, *p.adj* = 0.22 | τ = -0.09, *p* = 0.51, *p.adj* = 0.62 | τ = 0.55, ***p* < .01**, ***p.adj* < .01** | τ = 0.22, *p* = 0.12, *p.adj* = 0.31 |  |  |
| CVI Total | *r_s_* = -0.16, p = 0.51, p.adj = 0.62 | *r_pb_* = 0.24, *p* = 0.31, *p.adj* = 0.48 | *r* = 0.05, *p* = 0.84, *p.adj* = 0.84 | *r* = 0.33, *p* = 0.16, *p.adj* = 0.32 | *r* = 0.51, ***p* = 0.03**, *p.adj* = 0.19 | τ = -0.21, *p* = 0.20, *p.adj* = 0.38 |  |
| Epilepsy | τ = 0.29, *p* = 0.07, *p.adj* = 0.22 | *p* = 0.62, *p.adj* = 0.73^a^ | *r_pb_* = 0.55, ***p* < .01**, ***p.adj* = 0.03** | *r_pb_* = -0.34, *p* = 0.07, *p.adj* = 0.22 | *r_pb_* = 0.08, *p* = 0.71, *p.adj* = 0.75 | *r_pb_* = -0.34, *p* = 0.07, *p.adj* = 0.22 | *r_pb_* = 0.33, *p* = 0.16, *p.adj* = 0.32 |

τ Kendall; r_s_ Spearman; r_pb_ point-biseral ^a^Fisher’s exact test

## **Supplementary Table 5a.** Impact of Epilepsy on Adaptive Ability: Regression Assumptions

| Assumption | Test | Result |
| --- | --- | --- |
| Independence of Errors | Durbin-Watson | D-W = 2.31, *p* = .43 |
| Homoscedasticity | Breusch-Pagan | χ²(2) = 1.43, p = .23 |
| Normality of Residuals | Shaprio-Wilk | W = .98, p = .79 |

## **Supplementary Table 5b.** Impact of Epilepsy on Adaptive Ability: Regression Results

| Variable | B | SE | t | p | 95% CI |
| --- | --- | --- | --- | --- | --- |
| Constant | 89.63 | 3.57 | 25.11 | 2e-16 | [82.27, 96.98] |
| Epilepsy: Yes | 18.28 | 5.59 | 3.27 | 0.00313 | [6.77, 29.80] |
| R2 | 0.30 |  |  |  |  |
| Adj.R2 | 0.27 |  |  |  |  |

## **Supplementary Table 6a.** Impact of Epilepsy and Age on Adaptive Ability: Regression Assumptions

| Assumption | Test | Result |
| --- | --- | --- |
| Independence of Errors | Durbin-Watson | D-W = 1.95, *p* = .79 |
| Homoscedasticity | Breusch-Pagan | χ²(2) = 5.05, p = .08 |
| Normality of Residuals | Shaprio-Wilk | W = .96, p = .38 |
| Multicolliniarity | Variance Inflation Factors | All VIFs < 10 |

## **Supplementary Table 6b.** Impact of Epilepsy and Age on Adaptive Ability: Regression Results

| Variable | B | SE | t | p | 95% CI |
| --- | --- | --- | --- | --- | --- |
| Constant | 77.40 | 4.18 | 18.50 | 1.04e-15 | [68.76, 86.03] |
| Epilepsy: Yes | 11.00 | 4.80 | 2.81 | 0.032 | [1.04, 20.87] |
| Age | 1.56 | 0.39 | 3.97 | 0.00057 | [0.75, 2.38] |
| R2 | 0.58 |  |  |  |  |
| Adj.R2 | 0.54 |  |  |  |  |

## **Supplementary Table 7.** Summary of characteristics according to MRI abnormality within the BINGO *CASK*-related disorder group.

|  | MICPCH (n = 13)^a^ | | | Microcephaly (n = 9)^b^ | | | Normal (n = 2)^c^ | | | NA (n = 6)^d^ | | |
| --- | --- | --- | --- | --- | --- | --- | --- | --- | --- | --- | --- | --- |
|  | *M* | sd | Range | *M* | sd | Range | *M* | sd | Range | *M* | sd | Range |
| Age / years | 9.76 | 6.23 | 3.04-23.88 | 9.44 | 4.57 | 4.10-15.80 | 12.83 | 0.10 | 12.76-12.90 | 9.41 | 8.40 | 3.10-25.86 |
| Gender; n (% female) | 13 (100.00) | | | 7 (77.78) | | | 1 (50.00) | | | 5 (83.33) | | |
| VABS Composite^e^ | 40.15 | 15.25 | 20-68 | 43.62 | 14.24 | 22-61 | 59.00 | 21.21 | 44-74 | 50.4 | 21.73 | 20-75 |
| VABS Motor^e^ | 30.54 | 15.72 | 20-68 | 46.88 | 8.41 | 33-62 | 64.5 | 23.33 | 48-81 | 44.6 | 19.06 | 20-71 |
| CVI Total^f^ | 19.50 | 7.11 | 4-33 | 15.80 | 6.02 | 10-24 | 15.00 | 1.41 | 14-16 | 12.33 | 10.07 | 3-23 |
| Epilepsy; n (% present) | 7 (53.85) | | | 3 (33.33) | | | 0 (0.00) | | | 1 (20.00) | | |

^a^ Included participants who have reported MICPCH (note: one participant had CH/PH only, and one participant had MIC and CH only).

^b^ Included participants who have reported microcephaly (note: three participants have reported microcephaly and no available MRI data, one participant has reported microcephaly and unclear MRI data; four participants have reported microcephaly and normal MRI, and one participant has microcephaly and agenesis of corpus callosum).

^c^ Included participants whom have no reported microcephaly, or structural brain abnormalities, , either via MRI report, genetic report, or parent report.

^d^ Included participants for whom no brain abnormality data is available, either via MRI report, genetic report, or parent report.

^e^ Variable n - microcephaly n = 8, NA n = 5.

^f^ Variable n - MICPCH n = 10, Microcephaly n = 5, NA n = 3.

## **References**

1. Sparrow SS, Cicchetti DV, Saulnier, CA. Vineland adaptive behavior scales: Third edition (Vineland-3). Bloomington: NCS Pearson.; 2016.

2. Einfeld SL, Tonge BJ. The Developmental Behavior Checklist: The development and validation of an instrument to assess behavioral and emotional disturbance in children and adolescents with mental retardation. J Autism Dev Disord. 1995 Apr;25(2):81–104.

3. Constantino JN, Gruber CP. Social Responsiveness Scale–Second Edition (SRS-2). Torrance, CA: Western Psychological Services.; 2012.

4. Moss J, Oliver C. The Repetitive Behaviour Scale. Manual for administration and scorer interpretation. Birmingham, UK: University of Birmingham.; 2008.

5. Ortibus E, Laenen A, Verhoeven J, De Cock P, Casteels I, Schoolmeesters B, et al. Screening for Cerebral Visual Impairment: Value of a CVI Questionnaire. Neuropediatrics. 2011 Aug;42(04):138–47.

6. Ben Itzhak N, Vancleef K, Franki I, Laenen A, Wagemans J, Ortibus E. Visuoperceptual profiles of children using the Flemish cerebral visual impairment questionnaire. Dev Med Child Neurol. 2020 Aug;62(8):969–76.

7. Crotti M, Ortibus E, Mailleux L, Decraene L, Kleeren L, Itzhak NB. Visual, perceptual functions, and functional vision in children with unilateral cerebral palsy compared to children with neurotypical development. Dev Med Child Neurol. 2024 Aug;66(8):1084–95.

8. Hyman P, Oliver C, Hall S. Self-injurious behavior, self-restraint, and compulsive behaviors in Cornelia de Lange syndrome. Am J Ment Retard. 2002;107(2):146–54.

9. Bissell S, Oliver C, Moss J, Heald M, Waite J, Crawford H, et al. The behavioural phenotype of SATB2-associated syndrome: a within-group and cross-syndrome analysis. J Neurodev Disord. 2022 Dec;14(1):25.

10. Dunn W, Little LM, Dean E, Robertson S, Evans B. The Sensory Profile 2: Short Sensory Profile. 2nd ed. Pearson; 2014.

11. Dunn W. The Impact of Sensory Processing Abilities on the Daily Lives of Young Children and Their Families: A Conceptual Model: Infants Young Child. 1997 Apr;9(4):23–35.
